# Successful Management of Biliary Ascariasis in a High-Endemic Zone and Low-Resource Setting in Ethiopia

**DOI:** 10.1155/2022/8201398

**Published:** 2022-12-02

**Authors:** Yonathan Aliye Asfaw, Girum Tesfaye Asrat, Tesfanew Bekele Uddo, Bethlehem Aliye Asfaw, Ayush Anand, Helen Huang, Eden Demessie Firew, NagaSpurthy Reddy Anugu

**Affiliations:** ^1^University of Gondar Collage of Medicine and Health Sciences, Gondar, Ethiopia; ^2^Addis Ababa University, Addis Ababa, Ethiopia; ^3^B.P. Koirala Institute of Health Sciences, Dharan, Nepal; ^4^Royal Collage of Surgeons in Ireland, Dublin, Ireland; ^5^Mekelle University, Mek'ele, Ethiopia; ^6^Mediciti Institute of Medical Sciences, Hyderabad, India

## Abstract

Ascariasis lumbricoides is a roundworm that causes one of the most common soil-transmitted helminth infections worldwide. Ascariasis is typically found in the jejunum and transmitted through the intake of *Ascaris lumbricoides* eggs through food and water. Initially, ascariasis can cause pulmonary symptoms during the first 6–8 weeks of ingestion and can progress to serious complications of intestinal obstruction and hepatobiliary manifestations. Biliary ascariasis is a complication of ascariasis migration from the jejunum to the hepatobiliary tree and can present with a variety of symptoms ranging from biliary colic to more serious features such as acute cholangitis. Though the mortality rate is low, limited resources for management can make it challenging to manage.

## 1. Introduction

Approximately one-fourth of the world's population is infected by soil-transmitted helminths (STH) [1], caused by various parasites such as roundworms, whipworms, and hookworms [2]. These infections are widespread in economically poorer regions such as sub-Saharan Africa and south-east Asia [1–3], with Ethiopia alone accounting for approximately 29 million cases of STH among children in the year 2020 [1], which can be attributed to inadequate water sanitation, poor personal hygiene, and socioeconomic disparities [4]. Ascariasis is one of the commonly occurring STH infections [5] caused by *Ascaris lumbricoides* (roundworm), most commonly found in the jejunum [2, 6]. According to the Global Burden of Disease 2019 data, the estimated global prevalence of ascariasis was 594 per 100,000 people, with 2090 deaths and 754000 disability-adjusted life years [7]. It is transmitted through the intake of food and water contaminated with infectious *Ascaris lumbricoides* eggs [8]. Most patients are symptomatic, and the early phase pulmonary manifestations of Loeffler syndrome are uncommon in high endemic zones [3, 8]. Usually, the symptoms occur after 6–8 weeks of egg ingestion, which are often nonspecific [8] and can lead to complications such as intestinal obstruction [9], hepatobiliary and pancreatic involvement [10, 11].

Biliary ascariasis is one of the complications caused due to migration of *Ascaris* from jejunum to duodenum and through ampulla of Vater to the biliary tree [12], and is more common in women and children [10]. It can present as biliary colic, acute cholecystitis, and acute cholangitis [3, 10], and can be diagnosed based on clinical evaluation, stool examination, and imaging modalities such as plain X-ray, barium swallow, ultrasonography, endoscopy, and a computed tomography scan [13]. This condition can be treated by conservative management of clinical syndromes, administration of antihelminthic drugs, and endoscopy or surgical approaches if subsequent medical therapy fails [13, 14].

Though the mortality associated with ascariasis is low [15], the limited resources available for its management, coupled with its high endemicity in low-income countries, can make it challenging to manage. Herein, we report a rare case of obstructive jaundice secondary to biliary ascariasis in the high-endemic region of Ethiopia.

## 2. Case Report

**Figure 1:**
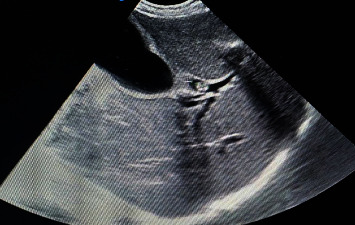
Ultrasound image of ascaris worm in the common bile duct, transverse section.

**Figure 2:**
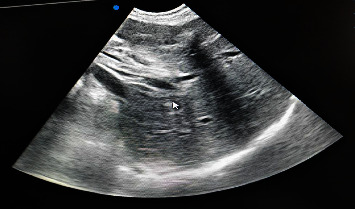
Ultrasound image of ascaris worm in the common bile duct, vertical section.

A 35-year-old woman presented to our hospital, Dilla University Referral Hospital, located in Dilla, southern Ethiopia, with abdominal pain of one-day duration. The pain was in the right upper abdomen, intermittent, colicky, and nonradiating, with no aggravating or relieving factors, and accompanied by anorexia and nausea. She denied any history of fever or vomiting. On physical examination, her vitals were normal, and icteric sclera was noted. Her abdominal examination revealed tenderness in the right hypochondriac region. Her laboratory investigations (Table 1) revealed neutrophilic leucocytosis, conjugated hyperbilirubinemia, and elevated alanine transaminase. On stool examination, ova of *Ascaris lumbricoides* were seen. Furthermore, ultrasonography (USG) of the abdomen revealed worms in the distal common bile duct with partial obstruction, a dilated gallbladder, and dilated right and left hepatic ducts (Figures 1 and 2).

**Figure 3:**
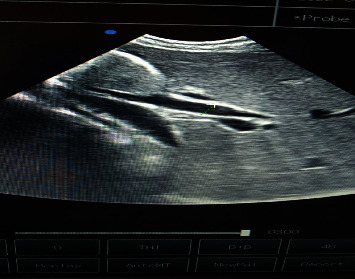
Ultrasound image showing normal common bile duct after worm exit.

Based on the high endemicity of ascariasis that is limited to the gastrointestinal tract in Ethiopia, the patients' history, clinical evaluation, stool examination, and USG report, a diagnosis of obstructive jaundice secondary to biliary ascariasis was made. To the best of our knowledge, this is the first case report in Ethiopia about biliary ascariasis, although it is quite common to see ascariasis cause disease manifestations limited to the small and large bowel. The management was done by administering intravenous normal saline, intravenous ceftriaxone 1 g bid, intravenous metronidazole 500 mg bid, intravenous hyoscine butyl bromide, and a single dose of 200 mg oral albendazole. A follow-up USG after 2 days did not reveal any worm in the common bile duct (Figure 3). The bile duct also didn't show any sign of dilation or stenosis after the worm exit. But it was not possible to check if the bile duct had developed any complications later on as the patient didn't show up for a follow-up USG scan. The hematologic profile of the patient returned to normal values (the total bilirubin came down to 1.2 mg/dl and the direct bilirubin decreased to 0.2 mg/dl) after the worm exit. After three days of hospital stay, the patient showed complete recovery and was discharged with a prescription for oral amoxicillin-clavulanate and metronidazole for one week.

## 3. Discussion

Biliary ascariasis is more prevalent in high-endemic zones and shows a female preponderance, with patients usually presenting in their mid-thirties [10]. There have been cases of ascariasis presenting as colic abdominal pain [16] and leading to obstructive jaundice [10]. Similarly, the patient was a female in her mid-thirties belonging to a high-endemic region of Ethiopia [1]. Her symptoms of colicky abdominal pain and conjugated bilirubinemia, coupled with elevated ALT and AST, most likely indicate obstructive jaundice due to acquired causes. Usually, we would expect a significant rise in ALP level in cases of obstructive jaundice, but this may not always be the case across all patients [17, 18].

Imaging of the biliary tree is indicated in patients presenting with right upper abdominal pain or jaundice [19–21]. Various imaging modalities, such as plain X-ray, barium swallow, ultrasonography, endoscopy, and computed tomography scanning can be used for imaging [13]. A plain X-ray of the abdomen may reveal a collection of worms contrasting against the bowel, known as the whirlpool effect, and a barium swallow can reveal elongated filling defects in the small intestine [22]. In addition, endoscopic retrograde cholangiopancreatography (ERCP) may be indicated in these patients as a diagnostic tool to determine the presence of worms in the CBD and can also act as a therapeutic modality with a high success rate [23]. Often, these patients are initially investigated with sonography, which can help detect various congenital and acquired abnormalities in the biliary tree [19–21]. Various studies and case reports have supported the use of USG in the diagnosis of biliary diseases [24–27]. In clinical guidelines, USG is already an established imaging modality for diagnosing biliary ascariasis and is crucial in low-resource settings due to its cost-effectiveness [28–31]. In our case, the patient was evaluated with USG, which suggested the presence of worms obstructing the CBD. In addition, stool examination revealed the presence of *Ascaris* eggs. Hence, a diagnosis of obstructive jaundice secondary to biliary ascariasis was made.

The management of biliary ascariasis can be done by conservative, endoscopic, and surgical approach [6]. These patients are hospitalized and treated with intravenous fluids, antispasmodic, and broad-spectrum antibiotics [10, 32, 33]. Antihelminthic drugs such as Albendazole, Mebendazole, and Pyrantel pamoate can be used to treat ascariasis [6]. A recent obervational study by Djune-Yemeli et al. concluded that a single dose of albendazole was insufficient to eliminate STH in high endemic regions [34]. However, a meta-analysis by Conterno et al. in 2020 revealed the cure rate of ascariasis by single-dose Albendazole and multiple-dose Albendazole as 93.2% and 94.3%, respectively [35]. Albendazole has also been concluded to have high efficacy and high safety profile for children and adults, making this treatment a viable option for patients [35]. Luckily, most patients recover with conservative management within 3 days and tend to be the best course of treatment for patients with milder symptoms [6, 10]. Nevertheless, for patients who are acutely sick or failed to respond to conservative management, ERCP or open exploration is the subsequent management option [6]. An endoscopic or surgical approach was not required in our case, as conservative management improved the patient's condition and resulted in the exit of worms from the common bile duct.

Biliary ascariasis will remain as a public health concern in high endemic and low-resource settings. Clinicians should approach biliary ascariasis with a high index of suspicion, as symptoms of abdominal pain and jaundice creates a wide variety of differentials that often cause the misdiagnosis of biliary ascariasis with other hepatobiliary pathologies, such as hepatocellular carcinoma or gallbladder stones. Though the development of efficient diagnostic tools have aided in the timely management of biliary ascariasis, it is common for tertiary hospitals to not have readily available tools such as an ERCP. Future research is warranted to determine the most efficacious diagnostic modality that is feasible for low-resource settings in the case of biliary ascariasis. Moreover, studies should investigate the sensitivity of biliary markers such as ALP, AST, and ALT in determining the presence of obstructive jaundice with biliary ascariasis to better guide informed clinical decisions.

There were several limitations to the case. We did not obtain GGT levels which would have further aided in the diagnosis of obstructive jaundice.

## 4. Conclusion

We report a case of a female patient in her mid-thirties presenting with right upper quadrant pain. She was diagnosed with obstructive jaundice secondary to biliary ascariasis based on endemicity, clinical evaluation, USG, and stool examination. This case highlights that biliary ascariasis should be suspected in patients presenting with symptoms of right upper abdominal pain in endemic areas. Also, abdominal ultrasonography can be a rapid, safe, noninvasive, and reasonably affordable imaging modality for diagnosing biliary ascariasis. Moreover, adequate conservative management with intravenous fluids, antispasmodics, broad-spectrum antibiotics, and antihelminthic treatment results in the successful management of these patients.

## Figures and Tables

**Table 1:** Laboratory investigations of the patient.

Investigation	Result	Reference range
Hb (g/dl)	13.4	11.0–17.5
MCV (fl)	83.9	79–94.8
MCH (pg)	30.4	25.6–32.2
MCHC (g/dl)	36.2	32.2–36.5
RBC (×10^6^/*µ*l)	4.41	3.93–6.08
WBC (×10^3^/*µ*l)	16.31	3.98–10.04
DLC (%)	N 89	N 34–71.1
	L 7.4	L 19.3–53.1
Platelet (×10^3^/*µ*l)	231	150–450

*Liver function test*		
Total serum bilirubin (mg/dl)	5.1	0.3–1.3
Direct bilirubin (mg/dl)	0.46	0.1–0.4
AST (IU/l)	92	12–38
ALT (IU/l)	106	7–41
ALP (IU/l)	57	44–147
AST/ALT	0.87	

Hb = hemoglobin concentration; MCV = mean corpuscular volume; MCH = mean corpuscular hemoglobin, MCHC = mean corpuscular hemoglobin concentration; RBC = red blood cell; WBC = white blood cell; DLC = differential leucocyte count; AST = aspartate transferase; ALT = alanine transaminase; ALP = alkaline phosphatase.

## References

[1] (2020). Soil-transmitted helminthiases. WHO. https://apps.who.int/neglected_diseases/ntddata/sth/sth.html.

[2] (2022). Soil-transmitted helminth infections. World health organization. https://www.who.int/news-room/fact-sheets/detail/soil-transmitted-helminth-infections.

[3] Jourdan P. M., Lamberton P. H. L., Fenwick A., Addiss D. G. (2018). Soil-transmitted helminth infections. *The Lancet*.

[4] Leung A. K. C., Leung A. A. M., Wong A. H. C., Hon K. L. (2021). Human ascariasis: an updated review. *Recent Patents on Inflammation & Allergy Drug Discovery*.

[5] Pullan R. L., Smith J. L., Jasrasaria R., Brooker S. J. (2014). Global numbers of infection and disease burden of soil transmitted helminth infections in 2010. *Parasites & Vectors*.

[6] Shah O. J., Zargar S. A., Robbani I. (2006). Biliary ascariasis: a review. *World Journal of Surgery*.

[7] (2019). Global burden of disease. *Ascariasis — Level 4 Cause*.

[8] Leder K., Weller F., Reddy N., Ascariasis - UpToDate, Ryan T., Baron L. (2022). https://www.uptodate.com/contents/ascariasis?search=ascariasis&source=search_result&selectedTitle=1%7E46&usage_type=default&display_rank=1.

[9] de Silva N. R., Guyatt H. L., Bundy D. A. P. (1997). Morbidity and mortality due to Ascaris-induced intestinal obstruction. *Transactions of the Royal Society of Tropical Medicine and Hygiene*.

[10] Khuroo M. S., Zargar S. A., Mahajan R. (1990). Hepatobiliary and pancreatic ascariasis in India. *The Lancet*.

[11] Khuroo M. S., Rather A. A., Khuroo N. S., Khuroo M. S. (2016). Hepatobiliary and pancreatic ascariasis. *World Journal of Gastroenterology*.

[12] Shah J., Shahidullah A. (2018). <b><i>*Ascaris lumbricoides*</i></b>: a Startling Discovery during Screening Colonoscopy. *Case Rep Gastroenterol*.

[13] Das A. K. (2014). Hepatic and biliary ascariasis. *Journal of Global Infectious Diseases*.

[14] Jethwani U., Singh G. J., Sarangi P., Kandwal V. (2012). Laproscopic management of wandering biliary ascariasis. *Case Reports in Surgery*.

[15] Khuroo M. S., Zargar S. A., Yattoo G. N. (2005). Ascaris-induced acute pancreatitis. *British Journal of Surgery*.

[16] Hassan Y., Rather S. A., Rather A. A., Banday M. K. (2021). *Ascaris lumbricoides* and the surgical complications: our experience from Medical College Hospital. *Irish Journal of Medical Science*.

[17] Gowda S., Desai P. B., Hull V. v., Math A. A. K., Vernekar S. N., Kulkarni S. S. (2009). A review on laboratory liver function tests. *Pan Afr Med J*.

[18] Hayat J. O., Loew C. J., Asrress K. N., McIntyre A. S., Gorard D. A. (2005). Contrasting liver function test patterns in obstructive jaundice due to biliary structures and stones. *QJM: International Journal of Medicine*.

[19] Yarmenitis S. D. (2002). Ultrasound of the gallbladder and the biliary tree. *European Radiology*.

[20] Rubens D. J. (2007). Ultrasound imaging of the biliary tract. *Ultrasound Clinics*.

[21] Foley W. D., Quiroz F. A. (2007). The role of sonography in imaging of the biliary tract. *Ultrasound Quarterly*.

[22] Reeder M. M. (1998). The radiological and ultrasound evaluation of ascariasis of the gastrointestinal, biliary, and respiratory tracts. *Seminars in Roentgenology*.

[23] Patra P. S., Das A., Ahmed S. M., Mitra S., Dhali G. K. (2021). Treatment response and long-term outcomes in biliary ascariasis: a prospective study. *Arab Journal of Gastroenterology*.

[24] Pinto A., Reginelli A., Cagini L. (2013). Accuracy of ultrasonography in the diagnosis of acute calculous cholecystitis: review of the literature. *Critical Ultrasound Journal*.

[25] Wertz J. R., Lopez J. M., Olson D., Thompson W. M. (2018). Comparing the diagnostic accuracy of ultrasound and CT in evaluating acute cholecystitis. *American Journal of Roentgenology*.

[26] Blackbourne L. H., Earnhardt R. C., Sistrom C. L., Abbitt P., Jones R. S. (1994). The sensitivity and role of ultrasound in the evaluation of biliary obstruction. *The American Surgeon*.

[27] Ghimire G., Lohani B., Pradhan S. (2005). Accuracy of ultrasonography in evaluation of level and cause of biliary obstruction: a prospective study - PubMed. *Kathmandu University Medical Journal*.

[28] Absi M., Qais A. M., al Katta M., Gafour M., Al-Wadan A. H. (2007). Biliary ascariasis: the value of ultrasound in the diagnosis and management. *Annals of Saudi Medicine*.

[29] Khuroo M. S., Zargar S. A., Mahajan R., Bhat R. L., Javid G. (1987). Sonographic appearances in biliary ascariasis. *Gastroenterology*.

[30] Gomez N. A., Leon C. J., Ortiz O. (1993). Ultrasound in the diagnosis of roundworms in gallbladder and common bile duct. Report of four cases. *Surgical Endoscopy*.

[31] Lentz B., Fong T., Rhyne R., Risko N. (2021). A systematic review of the cost-effectiveness of ultrasound in emergency care settings. *The Ultrasound Journal*.

[32] Khuroo M. S., Zargar S. A. (1985). Biliary ascariasis. *Gastroenterology*.

[33] Sultan Khuroo M., Ali Zargar S., Nabi Yattoo G. (1993). Worm extraction and biliary drainage in hepatobiliary and pancreatic ascariasis. *Gastrointestinal Endoscopy*.

[34] Djune-Yemeli L., Nana-Djeunga H. C., Lenou-Nanga C. G. (2020). Serious limitations of the current strategy to control Soil-Transmitted Helminths and added value of Ivermectin/Albendazole mass administration: a population-based observational study in Cameroon. *PLoS Neglected Tropical Diseases*.

[35] Conterno L. O., Turchi M. D., Corrêa I., Monteiro de Barros Almeida R. A. (2020). Anthelmintic drugs for treating ascariasis. *Cochrane Database of Systematic Reviews*.

